# 

**Published:** 1922-10

**Authors:** 


### GOVERNMENT PUBLICATIONS.

[Copies can be purchased through any bookseller or direct from H.M. Stationery Office, at Imperial House, Kingsway, W.C.2; 28 Abingdon Street, S.W.1; 37 Peter Street, Manchester; 23 Forth Street, Edinburgh; and 1 St. Andrew's Crescent, Cardiff. The prices quoted include postage.]

### Stationery Office Publications.

Scientific and Industrial Research.—Colloid Chemistry and its General and Industrial Applications. Fourth Report. 5s. 11d.

Health.—Model By-laws. Series IV, (*a*) Draft Form. New Buildings. 7½d.

Scottish Board of Health.—Inter-Departmental Committee on Milk. Minutes of Evidence. Law, Regulations and Procedure governing the Sale of Milk in Scotland, with Appendices. £1 10s. 8d.

Draft. Statutory Rules and Orders, 1922.—National Health Insurance. Approved Societies' Amendment. Regulations (No. 2), September 12, 1922. 1½d.

Ministry of Health.—Public Health and Medical Subjects Reports. No. 13. Bacteriological Studies. 2s. 8d.

Diphtheria.—Ministry of Health Memorandum on Supply of Diphtheria Antitoxin and on the use of the Schick Test. 1½d.

Sale of Food and Drugs Act.—Extract from Ministry of Health Report. 1s. 7d.

Ministry of Health. Circ. 335.—Milk and Dairies Amendment Act. 1922. 1½d.

Ministry of Health. Welfare of the Blind. Third Annua Report of the Advisory Committee. 7d.

### Statutory Rules and Orders.

(S.R.O. 901.) National Health Insurance. Discharged Seamen and Soldiers' Amendment Regulations, 1922 (August 10). 1½d.

(908.) Milk and Dairies (Consolidation) Act, 1915. (Commencement of Operation) Order, 1922. 1½d.

(Provl.) National Health Insurance, Medical Benefit (Amendment) Regulations. No. 2, 1922. 2½d.

### Public Acts.

Milk and Dairies Amendment (Ch. 54). 3½d.

Lunacy. (To amend the Law relative to Chancery Lunatics.) 2½d.

Criminal Law Amendment (Ch. 56). 2½d.

Local Government and Other Officers' Superannuation Act, 1922 (Ch. 59). 7d.

### RECENT APPOINTMENTS.

### Public Health.

Dr. Robert Veitch Clark, Medical Officer of Health for Croydon, to be Medical Officer of Health for Manchester.

Dr. Stella Churchill to be Assistant Medical Officer of Health for St. Pancras.

Dr. Cheeseman, late Assistant Medical Officer of Health for West Suffolk, to be Assistant Medical Officer of Health for Barrow-in-Furness.

Miss Marjorie Harris, late house physician at the Royal Hospital for Sick Children, Edinburgh, to be Assistant Medical Officer of Health for Oldham.

Mr. Hugh Stanford Bryan, late Assistant S.M.O. to the London County Council, to be Assistant School Medical Officer to the Norfolk Education Committee.

Dr. T. Philips Cole, Asst. S.M.O. to the West Sussex Education Committee, and formerly assistant house surgeon, casualty officer and house physician at Guy's Hospital, to be Medical Officer of Health for Bognor.

### Institutional.

Mr. A. G. Pike has succeeded the late Mr. George Sneath as treasurer to the Hendon (King Edward Memorial) Cottage Hospital.

Dr. Donald Paterson and Dr. Helen Mackay have been appointed as assistant physicians, and Dr. Enid M. Moore as anæsthetist, at the Infants' Hospital, Vincent Square, Westminster.

Dr. J. R. Currie, a senior medical officer of the Scottish Board of Health, has been appointed to the Chair of Preventive Medicine in Queen's University, Kingston, Ontario, Canada.

### OFFICIAL REPORTS RECEIVED.

**(Year to Dec. 31, 1921, unless otherwise stated) :—**

The Nightingale Fund. Year to December 25, 1921.

National Institute for the Blind. Year to March 31, 1922.

Association of Certificated Blind Masseurs. Year to June 30, 1922.

Borough of Middleton. Annual Report by Dr. S. Thos. Beggs, M.O.H. and S.M.O.

National Society for the Prevention of Cruelty to Children. Year to March 31, 1922.

Report on the Health of the City of Liverpool (1921). By Dr. E. W. Hope, O.B.E., Medical Officer of Health.

Report on the Sanitary Condition of Folkestone (1921). By Mr. M. G. Yunge-Bateman, M.R.C.S., Medical Officer of Health.

Burgh of Paisley; Report of the Public Health Department (1920 and 1921). By Mr. G. V. T. M'Michael, M.B., Ch.B., Medical Officer of Health.

The sixty-sixth Annual Report of the Registrar-Genera for Scotland, 1920, formerly published at £2 17s. 6d., has now been reduced in price to £1 10s. (post free £1 10s. 9d.). Copies are obtainable through any bookseller, or directly from H.M. Stationery Office, 23 Forth Street, Edinburgh.

### THE ROYAL SANITARY INSTITUTE.

New courses of training for Sanitary Officers, Health Visitors, School Nurses and Maternity and Child Welfare Workers begin at the Royal Sanitary Institute on September 27 and 29.

The Sanitary Officers' course comprises the subjects scheduled for the examination of the Institute and the Sanitary Inspectors' Examination Board. In addition to the lectures, students will visit public and private works illustrative of sanitary practice and administration, and the routine of an inspector's office work and duties will be demonstrated. The fee for the complete course is £5 5s.; for Part I. (September 27-November 15), £3 13s. 6d.; for Part II. (November 17-December 1), £2 2s. The subjects of lectures in Part I. include the Public Health, Factory and Workshop Acts; sanitary appliances, ventilation and warming; building sites and materials, and water supply and distribution; and in Part II. the laws, by-laws and regulations affecting the sale of food, the signs of health and disease in animals destined for food, and the hygiene of slaughterhouses, markets and other places where food is stored or handled.

The course for Meat and Food Inspectors consists of systematic practical training in the inspection of meat at a cattle market, with demonstrations on live cattle and sheep, slaughtering and dressing of animals, diseases of animals, methods of stalling, arrangements of markets and byres, &c.; a demonstration in fish inspection, and lectures on various foods, preservatives and the principles and practice of cold storage. Students can also attend the lectures in Part II. of the course for Sanitary Officers. The fee for the two months' complete course, beginning on October 6, is £5 5s., or without Part II., £4 14s. 6d.

The course for Women Health Visitors, School Nurses and Child Welfare Workers includes a comprehensive programme of 30 lectures. The hygiene of the home, the organisation and management of welfare centres and clinics, "health talks" to mothers, and the physical conditions affecting the health of elementary school children are a few of the subjects covered. The students will also attend six infant consultations under Dr. Eric Pritchard, and will visit infant welfare centres and schools for mothers. Sanitary appliances will be demonstrated at the Parkes Museum. The fee for the complete course is £5 5s.; for Part I. (September 29-November 13), £3 13s. 6d.; for Part II. (November 15-December 2), £2 2s. Candidates for the Health Visitors' and Child Welfare examinations must produce evidence of practical training and experience, including nursing. In July last the Ministry of Health issued a memorandum, naming the present certificate of the Institute as a qualification for salary grant for Health Visitors, provided that the holder has three years' training in a general hospital, or full training in a children's hospital. The Institute has decided, however, next year to require of all candidates three years' nursing training and the C.M.B., with attendance at a special course of training.

Full particulars of these courses and of the examinations of the Institute can be obtained from Mr. E. White Wallis, F.S.S., Director and Secretary, 90 Buckingham Palace Road, London, S.W.1.

---

### LECTURES ON INFANT AND CHILD WELFARE.

A course of lectures on Maternity, Child Welfare and School Hygiene is to be delivered on Wednesdays, at 4 o'clock, in the lecture hall of the Royal Institute of Public Health, 37 Russell Square, W.C.1, beginning on October 18. It is primarily intended for Fellows, Members and post-graduate students of the Institute, but others engaged in any form of public health service, or interested in medico-sociological problems, are invited to attend. No tickets of admission are required. The following is a list of the subjects and lecturers: October 18, "The Influence of Ante-natal Care upon Infant Mortality" (Professor Louise McIlroy); October 25, "The Organisation and Function of a Maternity Clinic" (Dr. G. E. Oates); November 1, "Pre-natal Hygiene and Problems of Maternity and Child Welfare" (Dr. W. M. Feldman); November 8, "The Problem of the Syphilitic Child" (Mrs. Margaret Rorke); November 15, "The Scientific Principles underlying Infant Feeding" (Dr. Eric Pritchard); November 22, "The Milk Problem, including the Use of Dried Milks" (Dr. Harold Scurfield); November 29, "Schoo Dental Clinics" (Mr. C. E. Wallis); December 6, "Medical Inspection in Secondary Continuation Schools" (Mr. C. W. Hutt); December 13, "Sunlight and Childhood" (Dr. C. W. Saleeby).

A course of twelve lectures to qualified practitioners on the management and feeding of infants and young children will be given by Dr. Eric Pritchard, physician in charge of the Infant Welfare Department, at the St. Marylebone General Dispensary, on Mondays and Thursdays at 6 p.m., from October 5 to November 13 inclusive. Students taking the course will be entitled to attend the infant consultations held by Dr. Pritchard, on Tuesdays and Fridays at 2 p.m., with cases in the wards at the Infants' Hospital, Westminster, and on Tuesdays at 11 a.m., and Thursdays at 3 p.m., at the Dispensary. On these days clinical demonstrations will be given as far as opportunity allows on the subjects referred to in the lectures. Students will also have opportunities of visiting the Nursery Training School, 3 Wellgarth Road, Golder's Green, on Saturdays at 3 p.m., and of seeing the same methods employed there in dealing with resident infants. Tickets (£2 2s. for the course) and further information can be obtained from the Secretary, St. Marylebone General Dispensary, 77 Welbeck Street, Cavendish Square, W.1.

The National Association for the Prevention of Infant Mortality announces two courses of elementary lectures on infant care, at Carnegie House, 117 Piccadilly, W.1. The first, especially intended for crèche nurses and probationers, will be given by Dr. Flora Shepherd, Medical Officer for Maternity and Child Welfare and Assistant Medical Officer of Health, Hornsey, on Thursdays, from 7.30 to 8.30 p.m., from October 5 to December 7. The other, for infant welfare workers, teachers, mothers, &c., will be given by a number of medical men and women on Mondays, from 6 to 7 p.m., from October 2 to December 11. Tickets (for the first course, 10s., for the second, 8s. 6d.; 1s. 3d. for a single lecture) must be applied for in advance, from the Hon. Secretary, N.A.P.I.M., 117 Piccadilly, W.1.

---

The Examining (Education) Board of the Institute of Hygiene gives notice that the next half-yearly examinations in hygiene and allied subjects will be held in London and various provincial centres on January 13, 1923. In addition to the examinations for the Certificate (Intermediate) and Diploma (Advanced) in general hygiene, certificate and diploma examinations will be conducted in the following special subjects, any of which may be taken separately: School hygiene, hygiene of the home, home nursing and first aid, mothercraft, food and dietetics, cooking (and foods), physical training, school nursing and health visiting. Candidates may be of either sex; those for the diploma must be over 18 years of age. Prospectus and syllabus can be obtained from the Secretary of the Institute of Hygiene, 33 Devonshire Street, London, W.1.

---

### HEALTH WEEK.

Health Week is to be held from October 8 to 14. Its object is to focus public attention for one week in the year on matters of health, and to arouse that sense of personal responsibility for health, without which all public work, whether by the Government or local authorities, must fall short of its aims. It is proposed that the dominant idea for 1922 shall be "Self-help in Health," and the consideration of what each individual can do for himself and his neighbour in securing a healthy life. The manner in which Health Week is observed in each district is determined by a local committee, which should include representatives of every public body and private society which is concerned with health, and of every agency which plays an important part in moulding public opinion. This committee may either take up a general health campaign or concentrate on some particular problem. The Executive Committee (90 Buckingham Palace Road, London, S.W. 1) advises the avoidance of questions of a doubtful or controversial nature, and of any undue harping on disease, preferring that stress should be laid on the saving of life and comparative immunity from disease which result from an efficient health administration and personal regard for health.

